# Analysis of Alphalactalbumin and Betalactoglobulin from the Rehydration of Bovine Colostrum Powder Using Cloud Point Extraction and Mass Spectrometry

**DOI:** 10.1155/2012/816180

**Published:** 2012-03-11

**Authors:** Fan Zhang, Xiaohua Qi, Mingqiang Zou, Jinfeng Li

**Affiliations:** Chinese Academy of Inspection and Quarantine, A3, North Gaobeidian Road, Chaoyang District, Beijing 100123, China

## Abstract

Alphalactalbumin (**α**-La) and betalactoglobulin (**β**-Lg) in the rehydration of bovine colostrum powder were successfully separated by cloud point extraction using a nonionic surfactant Triton X-114. The effects of different factors, including the surfactant concentration, sample volume, electrolyte, and pH were discussed. The optimized conditions for cloud point extraction of alphalactalbumin (**α**-La) and betalactoglobulin (**β**-Lg) can be concluded that the best surfactant is 1% (w/v) Triton X-114, 200 **μ**L of sample volume, 150 mmol/L NaCl, and 6% (w/v) sucrose. After cloud point extraction, the capillary electrophoresis is used to check the efficiency of the extraction procedure. The results had been effectively confirmed by the characterization with matrix-assisted laser desorption ionization time of flight mass spectrometry (MALDI-TOF MS).

## 1. Introduction

Bovine colostrum is an opaque white liquid which is a natural source of nutrition. Five main components in bovine colostrum are water, fats, proteins (e.g., caseins, albumins, and globulins), sugars (essentially lactose), and mineral salts. Since bovine colostrum spoils easily, it is sometimes processed into dairy products. The most durable form of bovine colostrum is bovine colostrum powder, which is produced by the dehydration of raw bovine colostrum. In developing countries, bovine colostrum powder is an important source of proteins in people's daily lives. Casein and whey proteins differ mainly in hydrophobicity and abundance. Casein is the major protein in cow's milk, and comprises about 80% of the total protein content of which the rest, 20%, are the whey or serum protein [1, 2]. Whey proteins display different structural characteristics and aminoacid compositions, with high nutritional level. Whey proteins contain alphalactalbumin (*α*-La), betalactoglobulin (*β*-Lg), immunoglobulin, lactoferrin, and bovine serum albumin [3]. Alphalactalbumin (*α*-La) and betalactoglobulin (*β*-Lg) are two kinds of whey protein in bovine colostrum and specifically produced in the mammary epithelial cells only during lactation [4]. As a monomer, alphalactalbumin (*α*-La) and betalactoglobulin (*β*-Lg) strongly bind calcium and zinc ions and may possess bactericidal or antitumor activity [5].

High-effect capillary electrophoresis (HPCE), namely, capillary electrophoresis (CE) is a rapid analytical technology. This technology already has applied to protein and aminofacid broadly for many years. Due to the appealing advantages in separation efficiency and resolution, small amounts of solvent and sample, CE have rapidly been applied for the assay in the protein separation field.

 For a long time, the capabilities of matrix-assisted laser desorption/ionization (MALDI) mass spectrometry for the rapid evaluation of proteins have been proved. Furthermore, it has also been tested to be a valid tool in the dairy industry. Although the technique does not determine the exact concentrations of bovine colostrum proteins, the relative proportions of several main bovine colostrum proteins can be identified. In this experiment, a portion of bovine colostrum powder was rehydrated with water. Another portion was separated with CPE method and detectable differences in their MALDI spectra were found.

The present work describes alphalactalbumin (*α*-La) and betalactoglobulin (*β*-Lg) in the rehydration of bovine colostrum powder were successfully separated by cloud point extraction using a nonionic surfactant Triton X-114. The separation was mainly based on their contrasting hydrophobicities, and our main goal was to achieve the highest concentration of alphalactalbumin (*α*-La) and betalactoglobulin (*β*-Lg) in the surfactant-poor phase obtaining high efficiency of CPE method. Since separation of these proteins using CPE depends on several variables, such as type and concentration of surfactant, pH, net charge, size and sample volume [6, 7], the optimization design was concluded in [8]. The matrix-assisted laser desorption ionization-time of flight mass spectrometry (MALDI-TOF MS) was used to confirm the separation of alphalactalbumin (*α*-La) and betalactoglobulin (*β*-Lg) from the rehydration of bovine colostrum powder.

## 2. Materials and Methods

### 2.1. Materials

 Bovine colostrum powder (Sunlife bovine colostrum powder) was purchased in a local Chinese market. All the water used in the experiment was Milli-Q deionized water (Millipore, Billerica, MA, USA.). Acetonitrile (HPLC grade) was obtained from SK Chemicals (Ulsan, Korea). The *α*-cyano-4-hydroxycinnamic acid (CHCA) was obtained from Applied Biosystems (Foster City, CA, USA). Trifluoroacetic acid (TFA) was obtained from Sigma-Aldrich (Steinheim, Germany). Tris (hydroxymethyl)-aminomethane, hydrochloric acid—HCl, sodium chloride—NaCl, isooctylphenyl ether (Triton X-114), and sucrose were obtained from Sigma-Aldrich (Steinheim, Germany).

### 2.2. Optimization Strategy

 There were four factors of optimization studied in this experiment. The factors were Triton X-114 concentration, sample volume, NaCl and sucrose concentration, and pH. The surfactant solution was prepared with 10 mmol/L Tris-HCl (pH 7.4), 150 mmol/L NaCl, and 6% (w/v) sucrose [8]. 200 *μ*L bovine colostrum sample was added to the surfactant solution, and the solution was homogenized again. Finally, the samples were centrifuged at 1780 ×g for 15 min. The surfactant poor phase was analysed in the next step. The temperature was 25° C in all experiments.

### 2.3. Capillary Electrophoresis (CE)

The CE was performed using a P/ACE MDQ capillary electrophoresis system (Beckman Coulter, Fullerton, CA, USA) under a normal polarity separation mode. A capillary (Beckman Coulter, Fullerton, CA, USA) with 50 cm effective length (60 cm total) and an inner diameter (I.D.) of 75 *μ*m was used with an applied voltage of 25 kV at 20^° ^C. Prior to use the capillary for the first time, a rinse for 1 min (30 psi) with 0.1 mol/L HCl was performed. A rinse for 10 min (30 psi) with the running buffer was performed then equilibrate the capillary for 10 min (25 kV). Finally a rinse for 10 min (30 psi) was performed The temperature of the separation capillary column was thermostated at 20° C.

Among each runs, the capillary was rinsed with 0.1 mol/L HCl for 0.5 min and then with the electrophoresis buffer for 1.5 min. The separation buffer was 50 mM Citrate buffer (pH 3.0), and the applied voltage is 25 kV. The electrophoresis buffers were filtered through 0.22 *μ*m filter before use. The samples were dissolved in 1 mL of buffer and filtered [0.22 *μ*m (Millipore)] for CE analysis. Samples were injected into the capillary using pressure at 0.5 psi for 10 s. The absorbance was measured at a wavelength of 214 nm for the detection of protein.

### 2.4. MALDI-TOF Sample Preparation

To prepare a saturated solution of CHCA matrix: 10 mg of the dry CHCA matrix was added to a tube containing 1 mL solution of water/acetonitrile/0.1% TFA (4/5/1, v/v/v). The tube was vortexed thoroughly for 1 minute and then centrifuged for 1 minute. 0.5 *μ*L of the sample/matrix (1/5, v/v) mixture was spotted per well on a stainless steel MALDI sample plate.

### 2.5. Mass Spectrometry

All the MALDI measurements were performed on a 4700 Proteomics Analyzer (Applied Biosystems, Foster City, CA, USA) equipped with a Nd:YAG laser (355 nm) operating at 200 Hz. Ions formed by a pulsed laser beam were accelerated to 20 Kilo Volts in positive ion linear mode with a 740 ns delay times. The Bin Size was set to 10 ns. The input bandwidth was set to 25 MHz. The acquisition method was set as follows: 60 subspectra were acquired on one spot and 25 shots were averaged per subspectrum, so one MALDI spectrum was the accumulated spectra of a total of 1500 shots per spot. For each sample, five spots were equally spotted and examined, and the acquisition method ran three times per spot. The final data were the trimmed means of the results of these 15 parallel tests as a whole. This instrument operating parameters were optimized for ion peaks corresponding to apomyoglbin (horse, m/z 16952), Thioredoxin (*E*. *coli*, m/z 11674), and insulin (bovine, m/z 5734).

## 3. Results and Discussion

### 3.1. CE Analysis Method and Results

**Figure 1:**
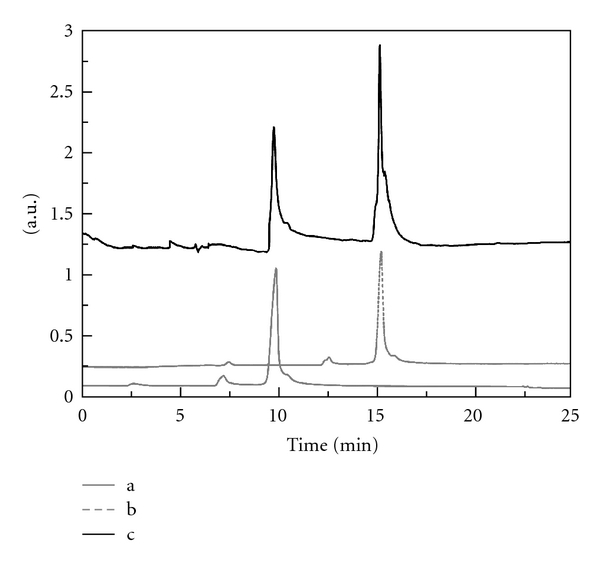
The electropherograms of standard substance and rehydration of bovine colostrum powder after cloud point extraction. There are alphalactalbumin (a), betalactoglobulin (b), and the sample (c).

To highlight the extraction of whey proteins from bovine colostrum and their separation from casein proteins by cloud point extraction, CE was used to identify *α*-lactalbumin and *β*-lactoglobulin extracted from the rehydration of bovine colostrum powder. The electropherograms of standard and cloud point extraction sample from the rehydration of bovine colostrum powder were shown in Figures 1(a) and 1(b) and Figure 1(c), respectively. The retention time of *α*-lactalbumin and *β*-lactoglobulin was about 10 and 15 min under the optimal separation condition. In Figure 1(c), there were only two whey protein peaks (*α*-lactalbumin and *β*-lactoglobulin). It was proved right with MALDI-TOF MS in the next experiment. From Figure 1(c), it is also proved the efficiency of the extraction procedure is successful.

### 3.2. MALDI-TOF MS Analysis

**Figure 2:**
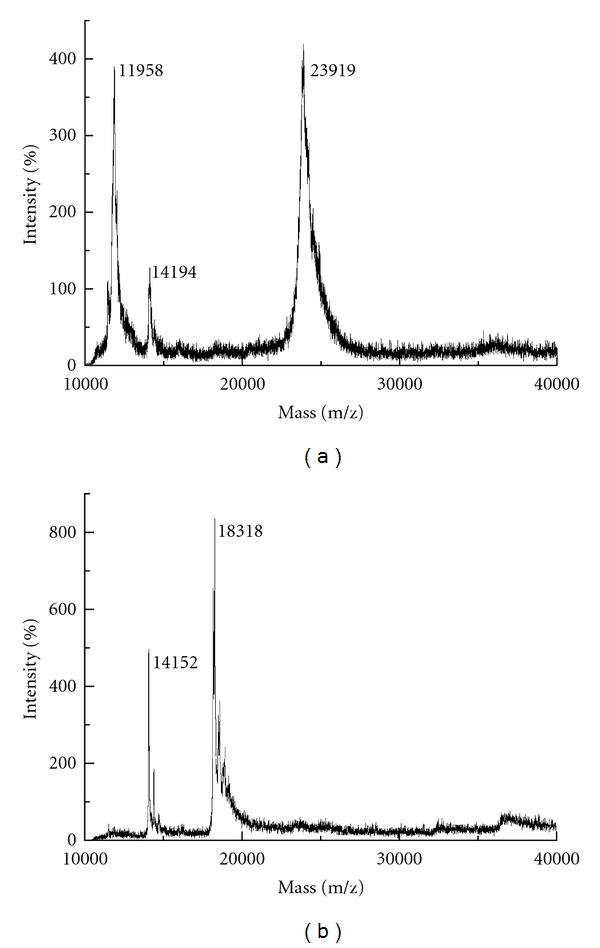
The MALDI-TOF mass spectra of bovine colostrum powder samples diluted with water rehydration (a) and bovine colostrum powder after cloud point extraction (b).

To highlight the extraction of whey proteins from bovine colostrum and their separation from casein proteins by CPE, MALDI-TOF MS to intact samples of the two protein fractions was applied. A simple sample treatment that only involved dilution of precipitated proteins was performed.  Figure 2(a) displays the mass spectra obtained, and it summarizes different proteins identified in the surfactant poor and water phases. The theoretical molar masses are in agreement with those previously described for whole cow bovine colostrum protein analyses by MALDI-TOF MS.

The mass spectra of Figure 2(a) show ions of ca.m/z11958 and 23919 corresponding to the *α*-casein and *β*-casein, respectively. Those ions in Figure 2(b) of higher m/z values correspond to *α*-lactalbumin (m/z14152) and *β*-lactoglobulin (m/z18318). Finally, the ion of ca.m/z 14152 in Figure 2(b) probably corresponds to species originating from lactose addition to *α*-lactalbumin. The main proteins identified in the surfactant poor phase (Figure 2(b)) were *α*-lactalbumin and *β*-lactoglobulin, which are fractions of the whey proteins [2]. Besides such proteins, *α*-casein and *β*-casein were also detected in the water phase. In fact, both last proteins also present amphiphilic character [4, 5], corroborating their presence in the surfactant poor phase.

## 4. Conclusion

CPE was efficiently employed to cow bovine colostrum samples being able to extract and separate whey from casein proteins using only 200 *μ*L of sample volume, sample volume without any sample pretreatment. Triton X-114 (1%, w/v), 150 mmol/L NaCl, and sucrose (6%, w/v) were used at pH 7.0 for protein separations. At such conditions, good partition coefficient was obtained, allowing the separation of *α*-lactalbumin and *β*-lactoglobulin (present in the surfactant poor phase) from casein proteins (present in the water phase) in only 15 min and with minimum costs, indicating that the proposed factorial design was successfully applied in the experimental domain employed and the main objective of this work attained. Although the partition coefficient was obtained, it is important to comment that some hydrophobic proteins were achieved in the surfactant poor phase and vice versa, as demonstrated through MALDI-TOF MS analysis. After cloud point extraction, the capillary electrophoresis was used to check the efficiency of the extraction procedure. The results had been effectively confirmed by the characterization with matrix-assisted laser desorption ionization time of flight mass spectrometry (MALDI-TOF MS). Finally, the adopted strategy could also be used for separating those low-abundant proteins in bovine colostrum after properly optimizing the CPE method for such task.
